# Root hairs enhance Arabidopsis seedling survival upon soil disruption

**DOI:** 10.1038/s41598-019-47733-0

**Published:** 2019-08-01

**Authors:** Hee-Seung Choi, Hyung-Taeg Cho

**Affiliations:** Department of Biological Sciences, Seoul National University, Seoul, 08826 Korea

**Keywords:** Abiotic, Plant ecology

## Abstract

Root hairs form a substantial portion of the root surface area. Compared with their nutritional function, the physical function of root hairs has been poorly characterised. This study investigates the physical role of root hairs of *Arabidopsis thaliana* seedlings in interaction of the root with water and soil and in plant survival upon soil disruption. Five transgenic lines with different root hair lengths were used to assess the physical function of root hairs. Upon soil disruption by water falling from a height (mimicking rainfall), long-haired lines showed much higher anchorage rates than short-haired lines. The root-pulling test revealed that a greater amount of soil adhered to long-haired roots than to short-haired roots. When seedlings were pulled out and laid on the soil surface for 15 d, survival rates of long-haired seedlings were higher than those of short-haired seedlings. Moreover, the water holding capacity of roots was much greater among long-haired seedlings than short-haired seedlings. These results suggest that root hairs play a significant role in plant survival upon soil disruption which could be fatal for young seedlings growing on thin soil surface with a short primary root and root hairs as the only soil anchoring system.

## Introduction

Evolution of roots enabled land plants to increase their size and consequently enhance their survival by strengthening the photosynthetic capability. An extensive root system not only plays a role in efficient water and mineral absorption from the soil but also provides mechanical support for the aboveground plant parts^1^. Root hairs, tubular protrusions from the root epidermis, increase the root surface area by approximately 2-fold, enabling the roots to reach fine soil particles tightly bound to essential nutrients^2^. Therefore, root hairs are considered important for nutrient uptake^3,4^ and show developmental plasticity in response to nutrient stresses^5^.

In contrast to their physiological role, the mechanical role of root hairs, such as soil anchorage, is poorly understood. In adult plants with a fully established secondary root system, lateral roots play a primary role in providing mechanical support to the plant^6^. Conversely, seedlings are usually equipped only with an elongated embryonic root and root hairs. Because most seedlings emerge on or near the soil surface^7–11^, they are prone to damage by unfavourable conditions such as exposure of the root after small-scale soil disruptions (e.g., rainfall) and other mechanical impacts on the soil surface.

We hypothesise that root hairs, as the only annexed structure of the seedling root, are important for soil anchorage and consequently seedling survival upon physical disruption of the soil surface. To test this hypothesis, we selected five transgenic *Arabidopsis thaliana* lines with different root hair lengths, and assessed whether root hairs contribute to soil anchorage, water holding capacity and survival rate, after the root was pulled out. Our results suggest that root hairs, especially long root hairs, are highly advantageous for the survival of seedlings upon the disruption of soil surface.

## Results and Discussion

### Root hairs constitute a larger portion of the root surface area in seedlings than in adult plants

Seedlings possess a simple root system comprising a short primary root and root hairs. By contrast, adult plants carry an extensive lateral root system. Therefore, we hypothesised that root hairs are critical for the physical function of seedling roots and examined whether the contribution of root hairs to the root surface area is greater in seedlings than in adult plants. First, we estimated the ratio of root hair surface area to the whole root surface area in young seedlings and adult plants with developing lateral roots. To calculate the ratio of root hair surface area from two different stages of Arabidopsis plants, we obtained the basic parameters of root hairs and primary and lateral roots from 15 previous studies and this study (Table 1; Supplementary Table S1). Young seedlings were 3–5-d-old after germination and had only a primary root with root hairs, whereas older plants were 10–14-d-old after germination and possessed lateral roots. Our results showed that root hairs account for ~61% and ~48% of the whole root surface area in young seedlings and older plants, respectively (Table 1), indicating a higher contribution of root hairs to the root surface area in seedlings than in older plants. A high proportion of root hairs in root surface area could be advantageous for the physical function in seedlings where lateral roots have not yet developed. Conversely, lateral roots play a primary mechanical role in adult plants^6^.

**Table 1:** Root parameters and calculation of the ratios of root hair surface area in seedlings and older plants.

Parameters	Seedlings (3–5 DAG)	Older plants (10–14 DAG)	References^a^
Root hair length^b^ (mm)	0.49 ± 0.11	0.38 ± 0.19	Supplementary Table S1
Root hair length of lateral root^b^ (mm)	N.A.	0.28 ± 0.16	Supplementary Table S1
Root hair diameter^c^ (μm)	9.9 ± 2.2		^26–29^
Primary root length^c^ (cm)	0.72 ± 0.4	70.15 ± 0.88	Supplementary Table S1,^30–33^
Primary root diameter^c^ (μm)	126.5 ± 36.8	145.3 ± 41.2	Supplementary Table S1,^27,28,34–39^
Total lateral root length^c^ (cm/seedling)	N.A.	5.93 ± 0.20	^32,33^
Lateral root diameter^c^ (μm)	N.A.	126.7 ± 47.1	Supplementary Table S1,^35–38^
Total length of root tip regions lacking root hair^c^ (mm/seedling)	0.95 ± 0.1	13.25 ± 1.7	Supplementary Table S1,^30,31,33,40^
Epidermal (H-) cell length^c^ (μm)	146.2 ± 36.1	191.5 ± 44.2	Supplementary Table S1,^27,34,35,39^
Epidermal (H-) cell length of lateral root^d^ (μm)	N.A.	137.4 ± 17.5	Supplementary Table S1
Root hair density (hair No./mm root)	47.5	43.6	
Total surface area of root hairs (mm^2^/seedling)	4.53	51.93	
Total surface area of root (mm^2^/seedling)	7.39	107.55	
Ratio of root hair surface area^e^ (%)	61.3	48.6	

^a^Among the accessions used in 15 references, 12 were Columbia-0 (Col-0)^26–30,32,33,35,37–40^ and three were Landsberg *erecta* (L*er*)^31,34,36^. Root hair densities were similar between Col-0 and L*er*^31,41–43^. Root hair lengths also were not significantly different (student *t* test, *P* > 0.05) in our observation. The means ± s.d. of root hair length are 0.41 ± 0.13 mm for Col-0 and 0.40 ± 0.13 mm for L*er* (n = 517–851 root hairs from 29–33 roots).

^b^Data represent mean ± s.d. (n = 159–423 root hairs from 10–11 roots).

^c^Data represent mean ± s.d. of the average values from each reference (n = number of reference).

^d^Data represent mean ± s.d. (n = 147 epidermal cells from 10 lateral roots).

^e^(Total surface area of root hairs/total surface area of root) × 100.

DAG, day after germination; N.A., not analyzed; No., number.

### Analysis of transgenic lines with different root hair lengths

To assess the importance of root hairs in root–soil interaction and seedling survival after soil disruption, we used five transgenic Arabidopsis lines with different root hair lengths. Expression of genes that regulate root hair length was analysed specifically in root hairs using the Arabidopsis root hair-specific promoter of the *EXPANSIN A7* gene (*ProE7*)^12,13^.

**Figure 1:**
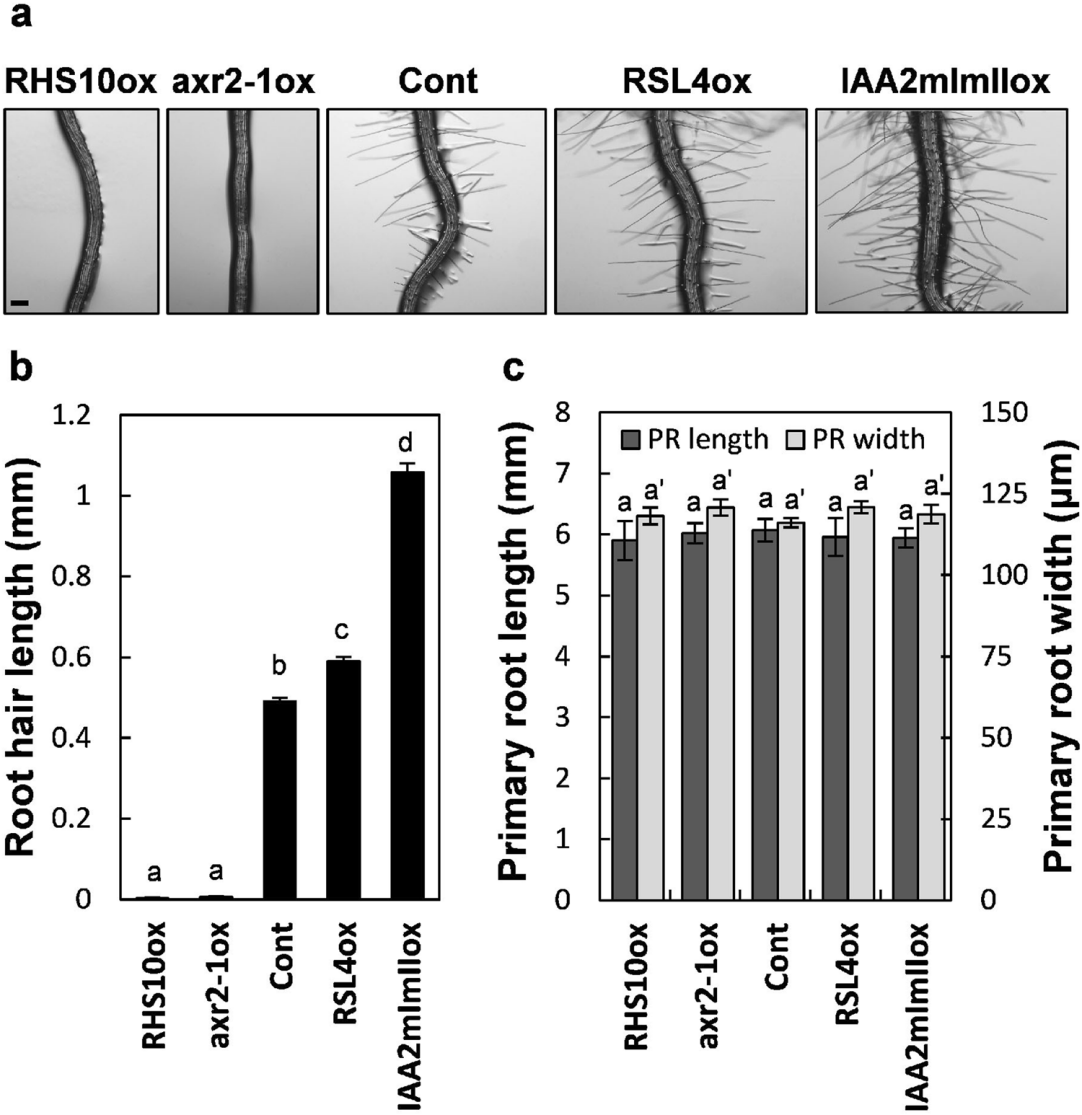
Transgenic lines with varying root hair lengths. (**a**) Representative root images of the control (Cont; *ProE7:YFP*) and transgenic lines overexpressing RHS10 (RHS10ox, *ProE7:RHS10*), axr2-1 (axr2-1ox, *ProE7:axr2-1*), RSL4 (RSL4ox, *ProE7:RSL4*), and IAA2mImII (IAA2mImIIox, *ProE7:IAA2mImII*). Bar is 100 µm for all. (**b**) Root hair length of the tested lines. Data represent mean ± s.e.m. (n = 151–511 root hairs from 13–39 roots from each line). (**c**) Primary root widths and lengths of the tested lines. Data represent mean ± s.e.m. (n = 12–36 roots from each line). Statistically significant differences are denoted with different letters (one-way ANOVA with Tukey’s unequal N-HSD *post hoc* test, *P* < 0.05, **b**,**c**).

Plants with root hairs of similar length as the wild type and expressing the *yellow fluorescent protein* (*YFP*) gene^14^ served as a control. Genes encoding ROOT HAIR SPECIFIC 10 (RHS10), a cell wall-associated receptor-like protein kinase, and axr2-1, the auxin-resistant form of Auxin/Indole-Acetic-Acid7 (IAA7), were expressed to obtain short root hair phenotypes^15,16^. Both these genes negatively modulate root hair tip growth. Root hair-specific overexpression of these genes (*RHS10ox* and *axr2-1ox*) greatly suppressed root hair elongation, with no noticeable effect on the primary root growth (Fig. 1).

To obtain long root hair phenotypes, we used transgenic plants expressing genes that encode ROOT HAIR DEFECTIVE SIX-LIKE4 (RSL4) and IAA2mImII proteins. The RSL4 is a basic helix-loop-helix (bHLH) transcription factor and a master regulator of *RHS* genes that regulate root hair morphogenesis^17–19^. The *RSL4* gene promoter is a direct target of auxin signalling and positively affects root hair elongation^17^. Transgenic seedlings with root hair-specific overexpression of *RSL4* (*RSL4ox*) showed ~20% longer root hairs than control seedlings (Fig. 1). The *IAA2mImII* gene harbours two mutations in the TOPLESS (TPL)-binding motif (mI) and auxin receptor-binding motif (mII) of *IAA2*, resulting in the loss of the repressive function of IAA2 in auxin signalling and resistance to auxin-mediated degradation. Because both mutations (mI and mII) in an Aux/IAA protein transform the repressor into an activator of auxin signalling by interfering with the recruitment of the TPL co-repressor, overexpression of *IAAmImII* in root hairs enhances root hair growth^20^. In this study, root hair length of transgenic seedlings overexpressing *IAA2mImII* (*IAA2mImIIox*) specifically in root hairs was approximately 2-fold greater than that of control seedlings (Fig. 1). The length and width of primary roots showed no significant differences among the five transgenic lines (Fig. 1c). These data suggest that root hair length is the only factor that affects the physical properties of the seedling root.

### Longer root hairs decrease seedling dislodging upon soil disruption

Rain has enormous kinetic energy sufficient to detach soil particles^21^ and dislodge young seedlings emerging from germinating seeds, thus exposing them to air. The survival rate of a seedling upon soil disruption depends on the expanse and strength of the seedling root system and/or the duration of exposure to dry conditions, until the root restores its gravitropical growth into the soil.

**Figure 2:**
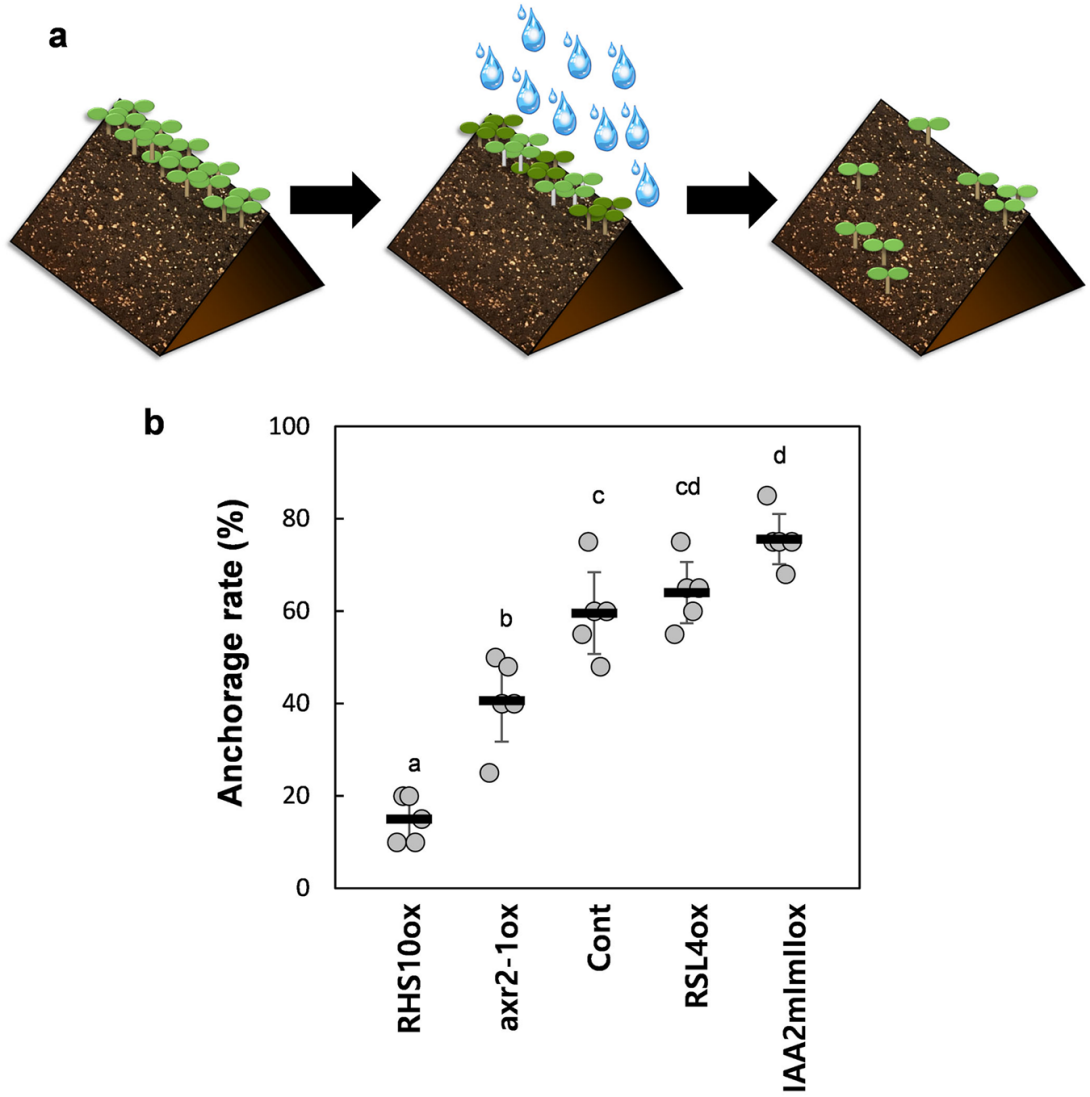
The anchorage test of seedlings after waterfall-mediated soil disruption. (**a**) Schematic images depicting soil disruption by water falling. For the real experimental setup images, see Supplementary Fig. S1. (**b**) Anchorage rates of control (Cont) and transgenic lines overexpressing RHS10 (RHS10ox), axr2-1 (axr2-1ox), RSL4 (RSL4ox), and IAA2mImI (IAA2mImIIox) after soil disruption by water falling. Data represent mean ± s.e.m. (n = 5 independent experiments). Statistically significant differences are denoted with different letters (one-way ANOVA with Tukey’s unequal N-HSD *post hoc* test, *P* < 0.05).

To mimic the effect of rainfall on soil and seedlings, we used a watering can to pour water on seedlings planted in a soil box; the box was tilted at an angle of 30° to facilitate soil disruption by water run-off (Fig. 2a). Anchorage rates were calculated by counting the number of seedlings that remained intact after soil disruption. The anchorage rate of *IAA2mImIIox* and *RSL4ox* long-haired seedlings was 76% and 64%, respectively, whereas that of control seedlings was 60% (Fig. 2b). By contrast, the anchorage rate of *axr2-1ox* and *RHS10ox* short-haired seedlings was 41% and 15%, respectively (Fig. 2b). These results suggest that root hairs enhance the survival of seedlings upon soil disruption by rainfall.

### Long-haired roots hold more soil than short-haired roots

**Figure 3:**
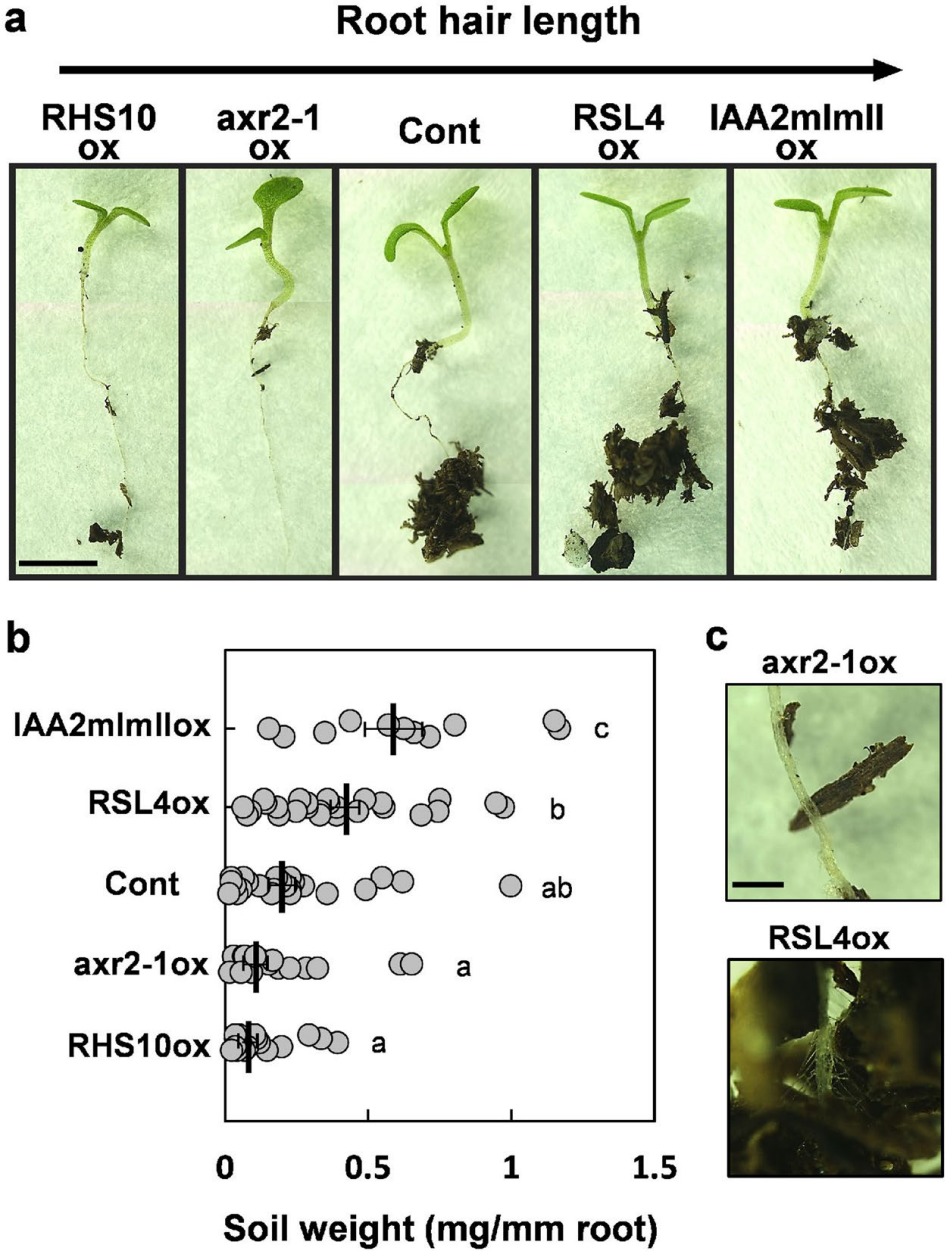
Soil-holding capacity of the seedling root. (**a**) Representative images showing soil held by the seedling root of control (Cont) and transgenic lines overexpressing RHS10 (RHS10ox), axr2-1 (axr2-1ox), RSL4 (RSL4ox), and IAA2mImI (IAA2mImIIox) after pulling out seedlings from soil. Bar is 5 mm for all. (**b**) Soil amount retained by the root after pulling out seedling. Data represent mean ± s.e.m. (n = 11–26 from each line). Statistically significant differences are denoted with different letters (one-way ANOVA with Tukey’s unequal N-HSD *post hoc* test, *P* < 0.05) (**c**) Magnified images of soil particles held by the root. Bar is 0.5 mm for all.

Higher survival rates of long-haired seedlings could be attributed to their higher soil holding capacity. To test this possibility, we estimated the soil holding capacity of roots by measuring the amount of soil held by the root after being pulled out from the soil. Our results showed that roots of long-haired lines (*IAA2mImIIox* and *RSL4ox*) held a 4–8-fold higher amount of soil than roots of short-haired lines (*axr2-1ox* and *RHS10ox*) (Fig. 3a,b). Analysis of the root–soil interface showed that clumps of soil particles adhered to root hairs of long-haired lines but barely to those of short-haired lines (Fig. 3c). This suggests that root hairs play a significant role in improving the soil holding capacity of seedling roots. Thus, root hairs function as a mechanical tool to hold soil, thus increasing the soil anchorage and survival of seedlings upon soil disruption.

### Root hairs enhance the seedling survival rate upon air exposure

**Figure 4:**
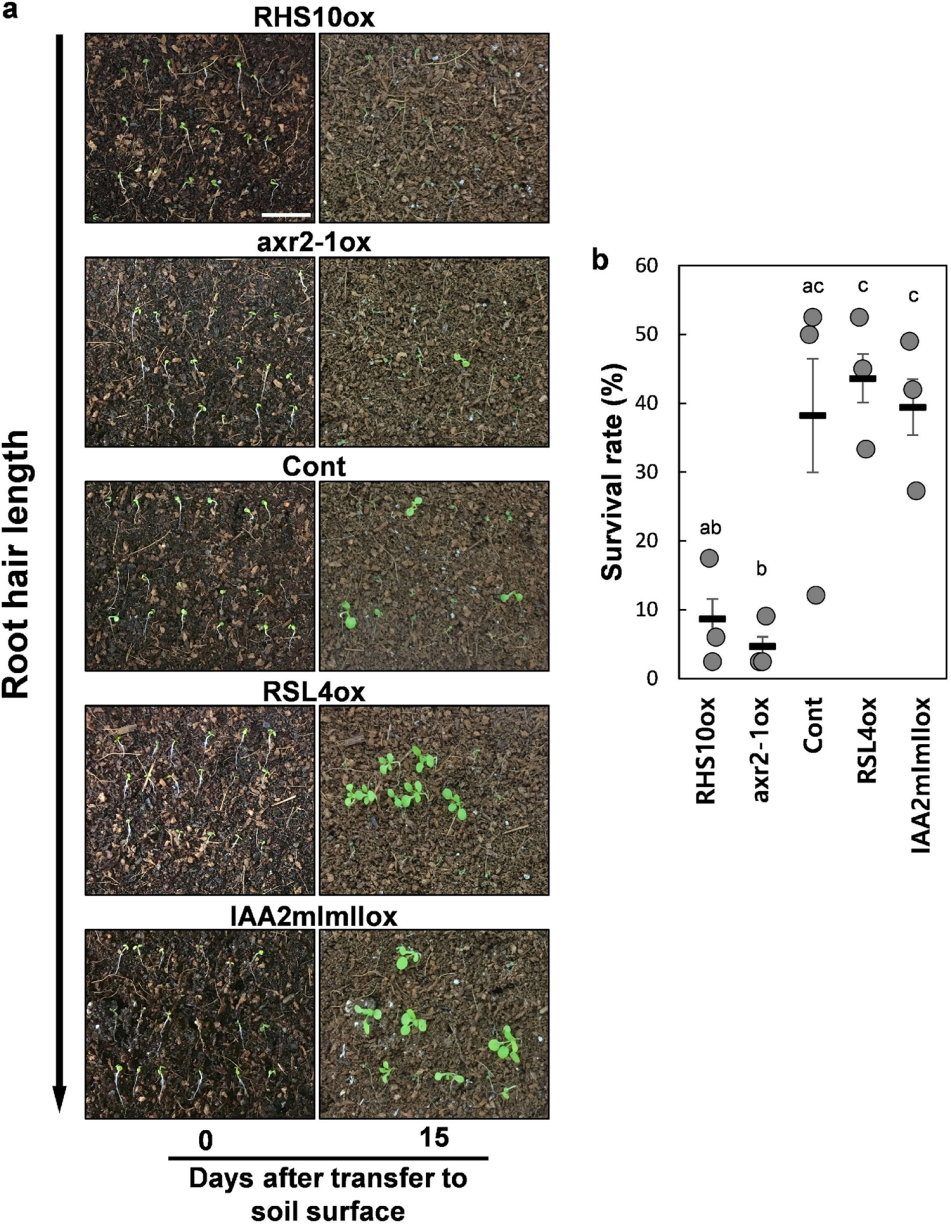
The survival test of naked seedlings on the soil surface. (**a**) Seedling images of control (Cont) and transgenic lines overexpressing RHS10 (RHS10ox), axr2-1 (axr2-1ox), RSL4 (RSL4ox), and IAA2mImI (IAA2mImIIox) after laying naked seedlings on the soil surface. Bar is 1 cm for all. (**b)** Survival rates of seedlings 15 days after laying on the soil surface. Data represent mean ± s.e.m. (n = 3 independent experiments). Statistically significant differences are denoted with different letters (one-way ANOVA with Tukey’s unequal N-HSD *post hoc* test, *P* < 0.05).

Because soil disruption can expose seedlings to air, which would dry the seedling root and eventually kill the seedling, we examined whether root hairs enable the seedling to survive under dry conditions following air exposure. To exclude the effect of soil held by seedling roots with different root hair lengths, we pulled the seedlings out of the agar medium, laid the naked seedlings on the soil surface and estimated their survival rates after 15 d. Our results revealed that longer root hairs were more advantageous for seedling survival in this test than shorter root hairs. While ~40% of the long-haired *IAA2mImIIox* and *RSL4ox* seedlings survived, less than 10% of the short-haired *axr2-1ox* and *RHS10ox* seedlings survived in this test (Fig. 4). This suggests that longer root hairs enhance the survival of seedlings exposed to dry conditions after soil disruption.

### Root hairs increase the water holding capacity of the root

**Figure 5:**
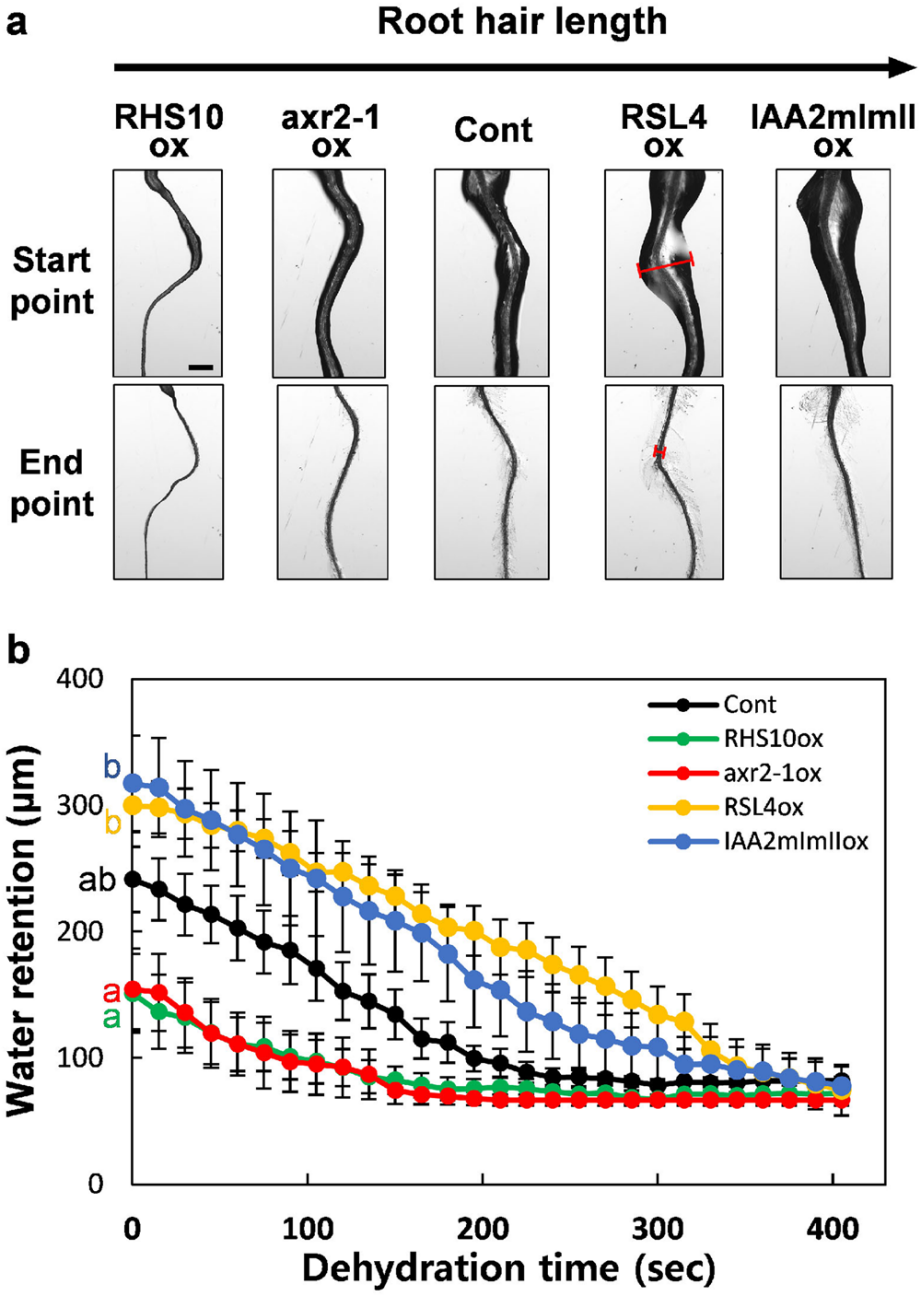
Water retention capacity of the seedling root. (**a**) Representative images showing water retention of the root from control (Cont) and transgenic lines overexpressing RHS10 (RHS10ox), axr2-1 (axr2-1ox), RSL4 (RSL4ox), and IAA2mImI (IAA2mImIIox) at the start point (immediately after pulling out the root from the agar medium) and the end point (when retained water has disappeared). Bar is 500 µm for all. Red bars in RSL4ox images indicate the water film widths including the root itself, which represents the capacity of water retention by the root. (**b**) The dynamics of water retention after pulling out the root from the agar medium. As shown in the images for RSL4ox in **a**, the widest width of the water film retained by the root was measured every 15 s after pulling out the root. Data represent mean ± s.e.m. (n = 25 roots from each line). Statistically significant differences at time 0 are denoted with different letters (one-way ANOVA with Tukey’s unequal N-HSD *post hoc* test, *P* < 0.05).

Until the gravitropic response of a dislodged seedling leads the root into the soil, the duration of exposure of the root to dry conditions is critical for seedling survival. If the root of a seedling pulled out of the soil can hold water for a long period of time, the seedling is expected to have a high chance of survival. To test this hypothesis, we pulled out seedlings from the agar medium and placed them on dry glass. A water film was formed around the root, which gradually dried over time. We measured the width of this water film, as a parameter of the water holding capacity of the root (Fig. 5a). Our results showed that, when the root was pulled out, long-haired roots exhibited much higher water retention than short-haired roots. Additionally, the width of water film showed that long-haired roots of *RSL4ox* and *IAA2mImIIox* seedlings retained approximately 2-fold more water than short-haired roots of *axr2-1ox* and *RHS10ox* seedlings (Fig. 5b). These data suggest that root hairs facilitate surface adhesion by liquid water, resulting in the formation of more water menisci around the root.

Furthermore, long-haired roots maintained the water film for a much longer period of time than short-haired roots (Fig. 5b). While the roots of *axr2-1ox* and *RHS10ox* seedlings retained the water film for 141–153 s, roots of *IAA2mImIIox* and *RSL4ox* seedlings retained the water film for 375–389 s (Fig. 5b). These results suggest that root hairs contribute to water retention by the root, thus increasing the chance of seedling survival when exposed to air upon soil disruption.

In soil holding-related tests as in Figs 2 and 3, the longest-haired *IAA2mimIIox* line slightly out-performed the other long-haired *RSL4ox* line. On the other hand, water holding-related tests revealed that the two long-haired lines performed similarly (Figs 4 and 5). It would be intriguing whether these results reflect that physical factors related with root hair length operate in different ways between soil holding and water holding. Regarding different physiological effects between transgenes in survival and physical tests, currently they are less likely because those root hair-specific transformants did not show noticeable phenotypes other than root hair length.

The control plant with wild-type level of root hair length performed similarly to or slightly less than the longer-haired *IAA2mImII* and *RSL4ox* lines in survival and physical capacity tests. These results might reflect that wild-type Arabidopsis root hairs have almost reached the optimal length to perform those physical functions.

While the nutritional function of root hairs has been extensively investigated, their physical function has not received much attention. In this study, we assessed the physical function of root hairs of Arabidopsis seedlings, where root hairs comprise a major portion of the root surface area. Therefore, we predict that the physical interaction of the root with soil is highly dependent on root hairs. Even mild environmental disruptions can be fatal to emerging seedlings that are merely laid on a thin soil surface. This study demonstrated that root hairs considerably enhance the water and soil holding capacity of roots, and these physical properties increase the survival rate of Arabidopsis seedlings upon soil disruption.

Although our results demonstrate some rudimentary insights into the role of root hairs in interaction with soil, they need to be interpreted with a certain limit because the native soil condition where Arabidopsis would grow should be different from our peat-based model soil condition.

Several lines of evidence in bryophytes suggest that root hair-like structures are important for preventing soil erosion. In bryophytes without a genuine root, rhizoids act as roots but hold root hair-like properties, in terms of development and cellular structure, except for their linear or branched multicellularity^4^. Unicellular rhizoids of a liverwort stabilise soil grains^22^. A recent geological investigation suggested that the beginning of terrestrial accumulation of mudrocks during the Ordovician–Silurian period (485–444 million years ago) coincides with land colonisation by bryophytes that possessed only root hair-like rhizoids to adhere to soil^23^. These studies, together with our results of root hair analyses, suggest that root hair-like unicellular structures are effective in increasing the interaction of seedling roots or bryophyte rhizoids with fine soil particles, thus preventing soil erosion.

## Methods

### Accession numbers

Genes studied in this article can be found in the Arabidopsis Genome Initiative or GenBank/EMBL databases under the following accession numbers: AT1G12560 (*EXPA7*), AT1G27740 (*RSL4*), AT1G70460 (*RHS10*), AT3G23030 (*IAA2*) and AT3G23050 (*AXR2*/*IAA7*).

### Plant material and growth conditions

*Arabidopsis thaliana* ecotype Columbia was used as the wild type and for the generation of all transgenic lines. To measure root parameters, transgenic T2 seeds were grown on 0.8% phyto-agar (Duchefa) medium containing 4.3 g L^−1^ Murashige and Skoog (MS) nutrient mix (Sigma-Aldrich), 1% sucrose, 0.5 g L^−1^ MES (pH adjusted to 5.7 using KOH) and 30 μg mL^−1^ hygromycin. A control line, *ProE7:YFP*^14^, was used for the analysis of transgenics on hygromycin-containing medium; hygromycin did not interfere significantly with root hair growth. To determine the water holding capacity of seedling roots, seeds were grown on 0.3% phyto-agar medium. To grow plants on soil, Sunshine Mix #5 (Sungro Horticulture) sieved through a 1-mm mesh was used. The same soil was used without sieving in other experiments. Plants were grown at 22 °C under a 16 h light/8 h dark photoperiod, ~130 μmol m^−2^ s^−1^ light intensity using fluorescent light bulbs (FHF 32SS-EXD, Kumho Electric) and 60–65% relative humidity, unless otherwise noted. All seeds were cold stratified at 4 °C for 3 d before germination.

### Plasmid construction

*ProE7p13M*, a modified *pCAMBIA1300-NOS* (Hyg+) binary vector including the root hair-specific *ProE7*^13^, was used to clone the *IAA2mImII* gene. To introduce the mI mutation in the TPL-binding domain I of IAA2 via site-directed mutagenesis, a mega-primer was generated by PCR using the primers 5′-TGATCCCGGGGCAATGGCGTACGAGAAAGT-3′ and 5′-GGTAATCCAAGAGCTAGCTCT-3′. The *IAA2mImII* sequence, which contains a mutation in the TPL-binding domain II of IAA2, was PCR amplified from the genomic DNA of the *IAA2* gain-of-function mII mutant using the mega-primer (containing the mI mutation) and 5′-CAAACCCGGGTCAGCTTCTCTGGATCATAAGG-3′. The amplified *IAA2mImII* sequence was inserted into the *Sma*I site of the *ProE7p13M* vector. The *ProE7:RSL4*, *ProE7:RHS10* and *ProE7:axr2-1* constructs and transgenic plants were generated as described previously^16,18^.

### Measurement of root parameters

Roots of 3–4-d-old and 10-d-old seedlings were observed under a stereomicroscope (M205 FA, Leica) at 12.5× or 40× magnification and photographed. Root parameters were measured from the digital images using the ImageJ 1.50 b software (National Institutes of Health, United States). Root hair length was measured as described previously^24^, with slight modifications. Lengths of eight consecutive root hairs protruding perpendicularly from each side of the root (16 root hairs in total) were measured. The width of the primary root was measured at three different points of the root. The length of root hair-forming epidermal cells was measured in the fully grown root hair region of the root.

### Calculation of the surface area of the root and root hairs

The surface area of the root and root hairs of seedlings and older plants (with lateral roots) was calculated based on our observations (Supplementary Table S1) and Arabidopsis root images published previously (Table 1). To calculate the surface area, roots and root hairs were considered as cylinders. The total root hair surface area of a plant was calculated from the length and diameter of a root hair and total number of root hairs in the root system. The hairless surface area of the root was measured by estimating the total root length, including the primary root and lateral roots in older plants. The total root surface area was calculated as the sum of the total root hair surface area and hairless root surface area. Because root hair density and length were different between the primary root and lateral roots, the total root surface area of older plant was estimated by summating the total primary root surface area and the total lateral root surface area. Then, the ratio of root hair surface area to the total root surface area was calculated. The total root hair number of a root was estimated from the root hair density (root hair number/root length excluding the length of root tip lacking root hairs), based on the observation that the Arabidopsis root radially contains eight root hair-forming epidermal cells (H-cells), 95% of which produce root hairs^25^. This method was adopted to include root hairs likely unseen under direct observation. The number of root hair-bearing H-cells in the whole root system was estimated from the root hair density [(8 H-cells × 0.95)/length of an epidermal H-cell] and total root length (excluding the hair-lacking root tip length), i.e., root hair density × total root length. Parameters including root hair diameter, primary root diameter, lateral root diameter, primary root length, lateral root length, root tip length and H-cell length were calculated from our observations (Supplementary Table S1) and by referring to images published in previous studies (Table 1).

### Waterfall-mediated soil disruption test

To conduct the survival test under waterfall-induced soil disruption, 4-d-old seedlings grown on agar medium were replanted in a rectangular box (44 cm length × 30 cm width × 8 cm height) filled with wet soil and grown for two additional days. To facilitate soil disruption, the soil box was inclined at 30° and 2 L of water was poured on the soil from a height of 0.1 m at a rate of 133 mL s^−1^ using a watering can with 138 apertures (1 mm in diameter) on the nozzle head (11 cm in diameter) (Supplementary Fig. S1). Seedlings that were not swept away by soil disruption were considered to survive the dislodging test. The anchorage rate of seedlings not-being-dislodged was calculated out of a total of 30 seedlings per line in each experiment.

### Soil-holding test

To determine the soil holding capacity of the root, 3-d-old seedlings grown on agar medium were planted in the soil, which enabled us to select the seedlings with similar root length for the test. After growing in the soil for 2 d, the seedlings were pulled out of the soil, and the total weight of the seedling (including the attached soil) was measured. To measure the weight of soil only, seedlings were washed with water to remove the soil, placed on tissue paper to absorb excess moisture and weighed. The seedling weight was subtracted from the total weight of seedling and soil, thus determining the weight of soil attached to the root, which was then normalised relative to the primary root length.

### Seedling survival test on the soil surface

To test the survival of seedlings on the soil surface, 5-d-old seedlings grown on agar medium were transferred to the surface of wet soil. Watering of soil was withheld for 14 d, and the number of surviving (green) seedlings was counted on day 15. Forty seedlings per line were used in each experiment.

### Water retention test

To determine the water holding capacity of the root, 3-d-old seedlings were pulled out from the agar medium (0.3%) and placed on dry glass under 20–22% relative humidity. Root images were obtained after every 15 s. The width of the widest region of the water film around the root, including the root width, was measured from each image until the water film dried completely, thus leaving the root naked (Supplementary Fig. S2).

Each experiment was replicated independently at least three times.

## Supplementary information

Supplementary Information

## Data Availability

All data generated or analysed during this study is available from the corresponding author on reasonable request.
