# Supplementary material for: Root hairs enhance Arabidopsis seedling survival upon soil disruption
Source: Sci Rep. 2019 Aug 1;9:11181. doi: 10.1038/s41598-019-47733-0 (PMC6671945; doi:10.1038/s41598-019-47733-0)
Supplement: Supplementary file 1 — Supplementary Information [file 41598_2019_47733_MOESM1_ESM.pdf]

Root hairs enhance Arabidopsis seedling survival upon soil disruption

Hee-Seung Choi and Hyung-Taeg Cho*

Supplementary Table S1. Root parameters in seedlings and older plants

**Parameters**[1]	**Seedlings (3-4 DAG)**	**Older plants (10-13 DAG)**
Root hair length of primary root (mm)	0.49±0.11	0.38±0.19
Root hair length of lateral root (mm)	N.A.	0.28±0.16
Epidermal (H-) cell length of primary root (μm)	180.6±32.4	181.4±47.4
Epidermal (H-) cell length of lateral root (μm)	N.A.	137.4±17.5
Primary root diameter (μm)	116.1±8.5	134.4±10.9
Lateral root diameter (μm)	N.A.	85.0±47.1
Root tip length lacking root hair (mm)	0.87±0.14	N.A.

[1] Data represent mean ± s.d. (n = 159-423 root hairs, 45-147 epidermal cells from 10-11 seedlings)

N.A., not analyzed

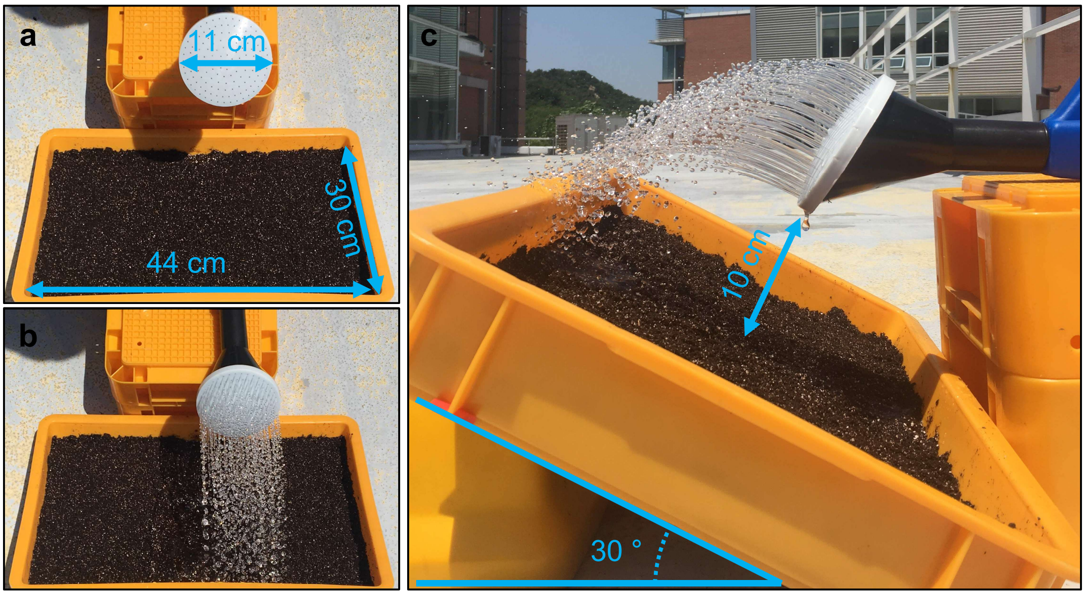

Supplementary Figure S1. Images showing the waterfall-mediated soil disruption experiment. (**a**) The top view of the setting before watering. (**b**) The top view of the setting during watering. (**c**) The side view of the setting during watering.

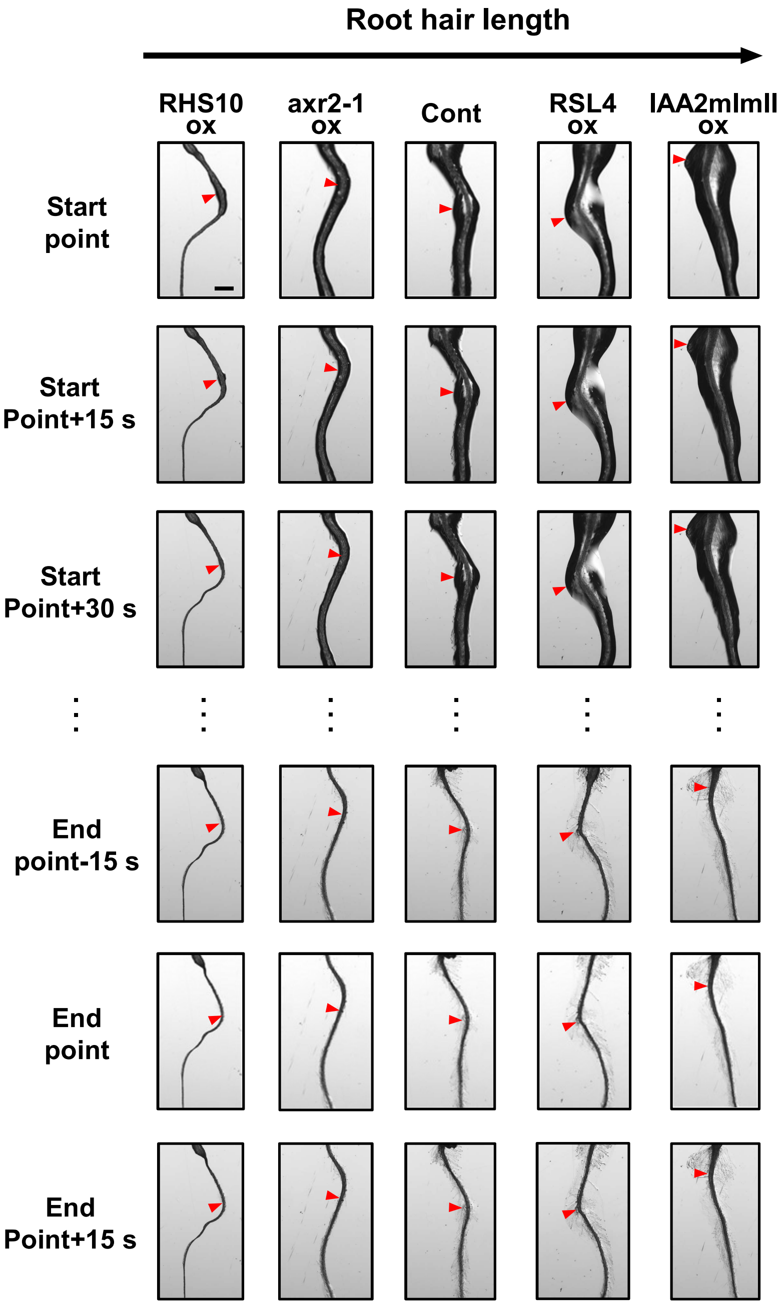

Supplementary Figure S2. Time-lapse images of the root showing the change of water retention. The root images are from control (Cont) and transgenic lines overexpressing RHS10 (RHS10ox), axr2-1 (axr2-1ox), RSL4 (RSL4ox), and IAA2mImI (IAA2mImIIox). Root images of the start point were obtained immediately after pulling out the root from the agar medium. The end point indicates the time point when no more decrease in water film width occurs as shown in Fig. 5. Arrow heads indicate the position where the water film width was measured. Bar is 500 μm for all.
